# Theoretical Investigation on the Selective Adsorption of ReO_4_^−^ by Functional Monomers: The Role of Hydrogen Bonding and Anion–Heterocycle Interactions

**DOI:** 10.3390/ijms27093881

**Published:** 2026-04-27

**Authors:** Jiongyao Wu, Bo Wang, Yang Gao

**Affiliations:** 1 Department of Physics, University of Duisburg-Essen, Lotharstrasse 1, D-47057 Duisburg, Germany; 2 School of Science, Northeast Electric Power University, Jilin 131200, China; 3 Institute of Fundamental and Frontier Sciences, University of Electronic Science and Technology of China, Chengdu 611731, China

**Keywords:** density functional theory (DFT), anion-heterocycle interaction, anion recognition, hydrogen bonding, selective adsorption

## Abstract

Understanding the adsorption mechanisms of anions onto functional monomers is crucial for various applications in environmental remediation and chemical separation. In this study, we investigated the interactions of ReO_4_^−^, Cl^−^, SO_4_^2−^, and F^−^ with several organic functional monomers featuring aliphatic chains, cyclic saturated or unsaturated rings, and NH/NH_2_ functional groups through density functional theory calculations. Employing a rigorous multilevel optimization strategy, we explored the geometric and energetic features of their complexes, focusing on hydrogen bonding and anion–heterocycle interactions. Our results highlight the strong affinity of ReO_4_^−^, Cl^−^, SO_4_^2−^ for amine-type functional monomers, while also revealing the distinct interaction patterns of F^−^ with aromatic rings containing nitrogen. This comprehensive analysis elucidates diverse binding mechanisms, providing insights into designing effective adsorbents for selective anion capture.

## 1. Introduction

Rhenium (Re), among the rarest and most valuable metallic elements, is crucial for applications spanning alloys, catalysis, aerospace technology, and nuclear medicine, owing to its exceptional physical properties: ductility, high density, elevated melting point, and robust resistance to heat, abrasion, wear, and corrosion [1,2,3,4]. Unlike many metals, Re does not occur naturally in a singular mineral form, posing challenges to its extraction and utilization [5,6,7,8]. ReO_4_^−^, a principal chemical form of Re, shares a tetrahedral structure and akin ionic radius with pertechnetate (TcO_4_^−^), making it a non-radiating structural analog of TcO_4_^−^ [9,10]. Pertechnetate, highly soluble (11.3 mol·L^−1^ at 293 K) with minimal complexation and rapid migration, poses significant environmental and health hazards by permeating the food chain [11,12,13,14]. Therefore, understanding the properties and interactions of ReO_4_^−^ is crucial for applications in environmental remediation, nuclear waste management, and medical diagnostics and therapy.

Various methods have been employed to extract and recycle ReO_4_^−^, including ion exchange, precipitation, solvent extraction, and adsorption, each presenting distinct advantages and challenges [15]. Among these methods, adsorption stands out for its environmental compatibility, offering high efficiency, simplicity in operation, cost-effectiveness, and substantial recovery potential for dilute solutions. However, the industrialization of adsorption processes faces formidable hurdles due to the stringent conditions required for optimal performance. Despite extensive exploration of various adsorbents such as activated carbon [16], chitosan [17], cellulose [18], β-cyclodextrin [19], silica [20], titanium dioxide [21], and aminocarboxylate adsorbents [22], achieving scalable and economically viable solutions remains a critical challenge. These materials, while promising in laboratory settings, often require further refinement to withstand the complexities of real-world applications. Key research focuses include enhancing selectivity, improving adsorption capacity, and optimizing regeneration methods to ensure sustainable and efficient ReO_4_^−^ recovery.

The oxygen atoms in ReO_4_^−^ can form hydrogen bonds with the hydrogen atoms in the substrate, establishing strong interactions between the adsorbent and ReO_4_^−^. Consequently, functional groups capable of forming hydrogen bonds, such as –NH and –CH, play a crucial role in enhancing adsorption properties [23,24,25,26,27,28,29]. Despite its pivotal role, the experimental determination of the adsorption mechanism poses significant challenges due to its dynamic and complex nature. Density functional theory (DFT) emerges as a powerful tool for elucidating adsorption mechanisms and screening functional monomers with high precision and computational efficiency [30,31,32,33]. By simulating molecular interactions at the atomic level, DFT provides insights into the specific roles of hydrogen-bonding groups in facilitating ReO_4_^−^ adsorption. However, to date, systematic investigations into the detailed adsorption mechanisms between hydrogen-bonded monomers and ReO_4_^−^ are lacking in the literature. This study addresses this gap by employing DFT to comprehensively analyze the adsorption mechanism of ReO_4_^−^ with monomers bearing hydrogen bonding functional groups. Through computational modeling, we aim to unravel the intricacies of hydrogen bond interactions, providing a deeper understanding of their impact on adsorption efficiency and selectivity.

To deepen our understanding of ReO_4_^−^ adsorption mechanisms using functional monomers, this study employs density functional theory (DFT) calculations. We systematically selected and analyzed a series of promising adsorption monomer structures, investigating their interactions with ReO_4_^−^ as well as Cl^−^, F^−^, SO_4_^2−^. These additional anions were selected as representative competing species commonly found in industrial wastewater and radioactive waste streams, allowing for a comprehensive evaluation of the adsorption selectivity for ReO_4_^−^ in the presence of ions with varying charges, sizes, and hydration energies. By assessing the influence of hydrogen bonding and anion-heterocycle interactions on adsorption efficiency and selectivity, our computational simulations elucidated specific mechanisms governing these associations and enhanced our comprehension of their pivotal role in increasing anion adsorption capacity. This research aims to provide theoretical insights and a scientific foundation for the development of efficient, selective, and sustainable anion adsorbents.

## 2. Results and Discussion

**Figure 1:**
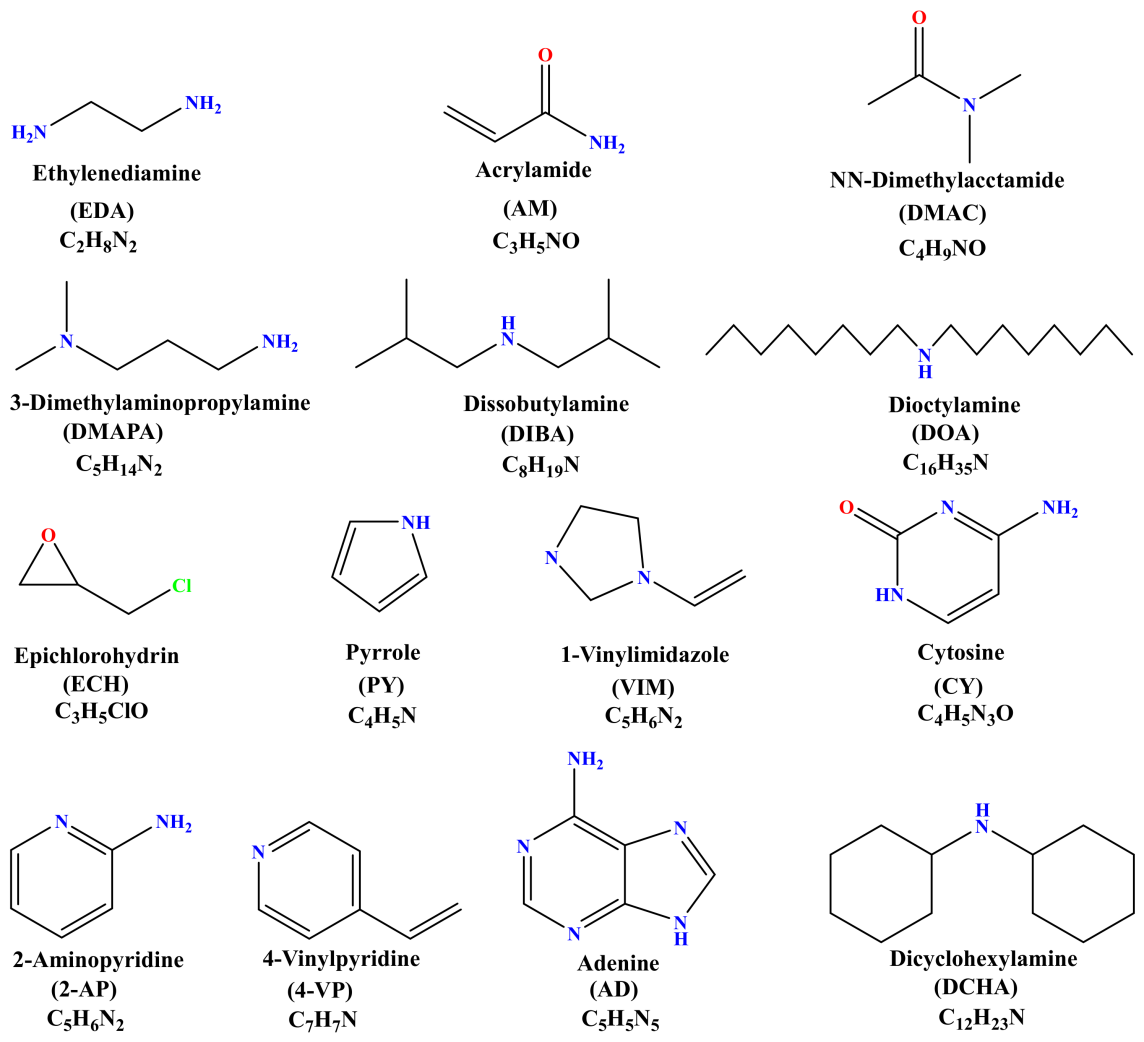
The fourteen functional monomers are studied in this paper.

To ensure a reliable physical starting point for the adsorption studies, we first verified the electronic stability of ReO_4_^−^ under relativistic perturbations. Comparative analysis of scalar relativistic and spin-orbit coupling effects confirms that the fundamental electronic structure of the oxo-anion remains remarkably robust, with the HOMO-LUMO gap exhibiting only a negligible fluctuation of 0.06 eV. Detailed energy level splitting schemes and relativistic benchmarks are provided in the Supplementary Material (see Figure S1). With the electronic stability of the adsorbate confirmed, the investigation shifts to the role of the functional monomers. According to the types of N groups and their chemical environments, we classified the fourteen functional monomers into three categories: amine types, nitrogen-containing ring types, and nitrogen-free ring types. The amine types include primary amines (such as Ethylenediamine (EDA), Acrylamide (AM), and 3-Dimethylaminopropylamine (DMAPA)), secondary amines (such as Dioctylamine (DOA), Diisobutylamine (DIBA), and Dicyclohexylamine (DCHA)), and tertiary amines (such as N,N-Dimethylacetamide (DMAC)). These functional groups, represented as NH_2_, NH, or NR_2_, impart them with the ability to act as basic proton acceptors in chemical reactions. Among these, the varying degrees of alkyl substitution significantly influence their basicity and steric accessibility. For instance, the long-chain symmetric secondary amines like DOA and DCHA provide a balance between hydrophobic stabilization and focused hydrogen-bonding sites, which are essential for coordinating with large tetrahedral anions like ReO^4−^. The nitrogen-containing ring types consist of Adenine (AD), 1-Vinylimidazole (VIM), Cytosine (CY), 4-Vinyl Pyridine (4-VP), 2-Aminopyridine (2-AP), and Pyrrole (PY). These monomers are characterized by their rigid pi-conjugated systems, with at least one nitrogen atom distributed within the ring structure or attached to the ring. The integration of nitrogen into aromatic frameworks not only modulates the electron density of the rings but also introduces the potential for multifaceted interactions, such as dual-site hydrogen bonding and anion-pi stacking, which distinguish them from aliphatic amines. The nitrogen-free ring type includes Epichlorohydrin (ECH), representing a control group to evaluate the necessity of nitrogen-containing groups in the adsorption process. The structures of these monomers are depicted in Figure 1.

**Scheme 1:**
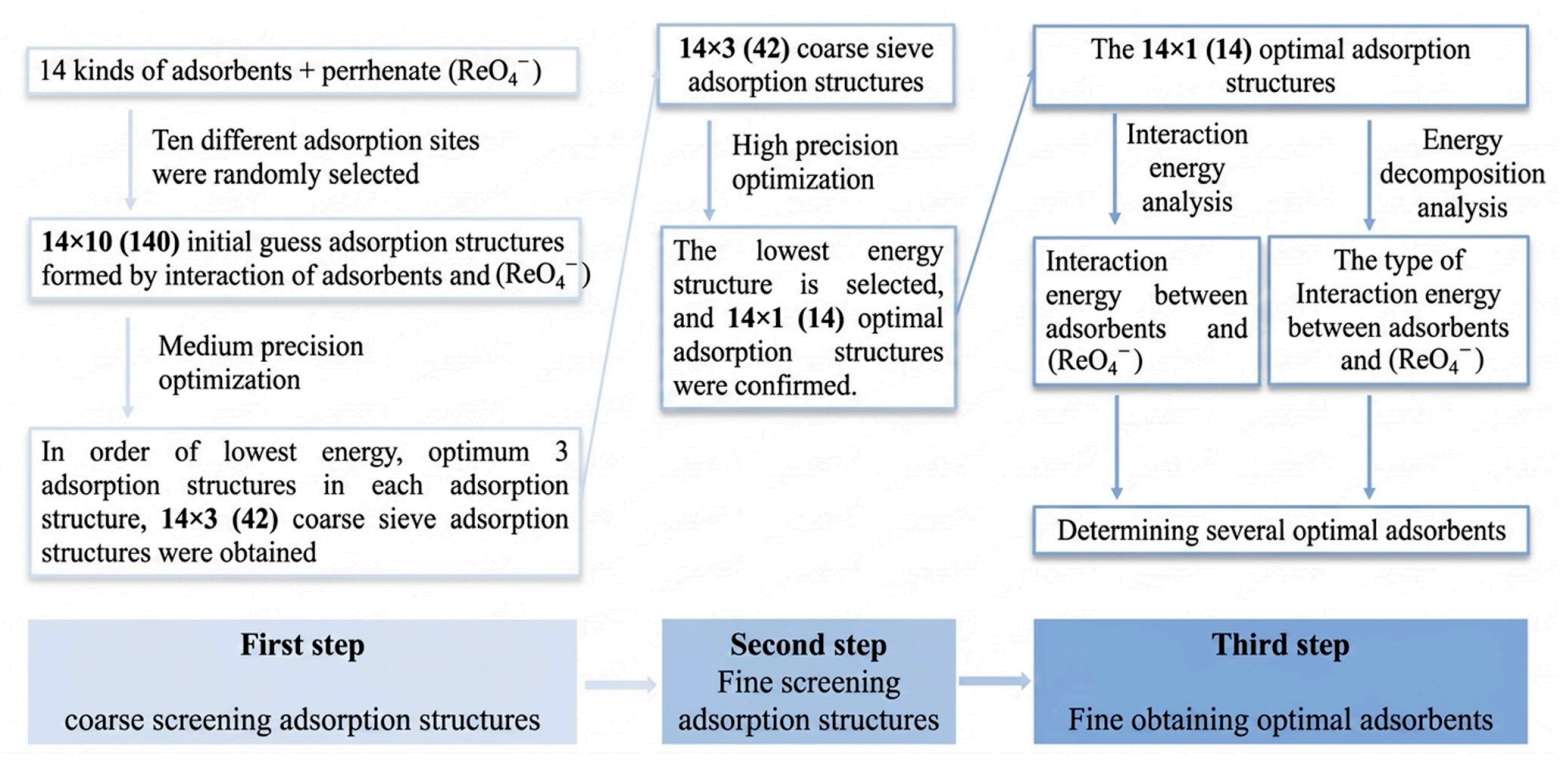
Multilevel optimization strategy for the interaction between functional monomers and ReO_4_^−^.

To accurately determine the geometric and energetic characteristics of the adsorption structures formed by various molecules interacting with ReO_4_^−^, ensuring the reliability and accuracy of computational results, we employed a multilevel optimization strategy (as depicted in Scheme 1). To ensure the reliability of our structural predictions, we employed a multi-stage screening strategy (detailed in Section 4). The initial stochastic sampling generated 140 candidate adsorption configurations for the 14 functional monomers, from which the three lowest-energy structures for each system were selected for further refinement. This rigorous process yielded 42 distinct adsorption complexes for high-precision optimization. Upon final structural refinement, 14 optimal adsorption complexes were identified, representing the most stable binding sites for each monomer. The energy rankings and geometric parameters for these intermediate and final structures are summarized in Table S1 and Table S2, respectively. The high consistency between our initial energy-based screening and the final refined geometries underscores the robustness and accuracy of our structural models.

**Figure 2:**
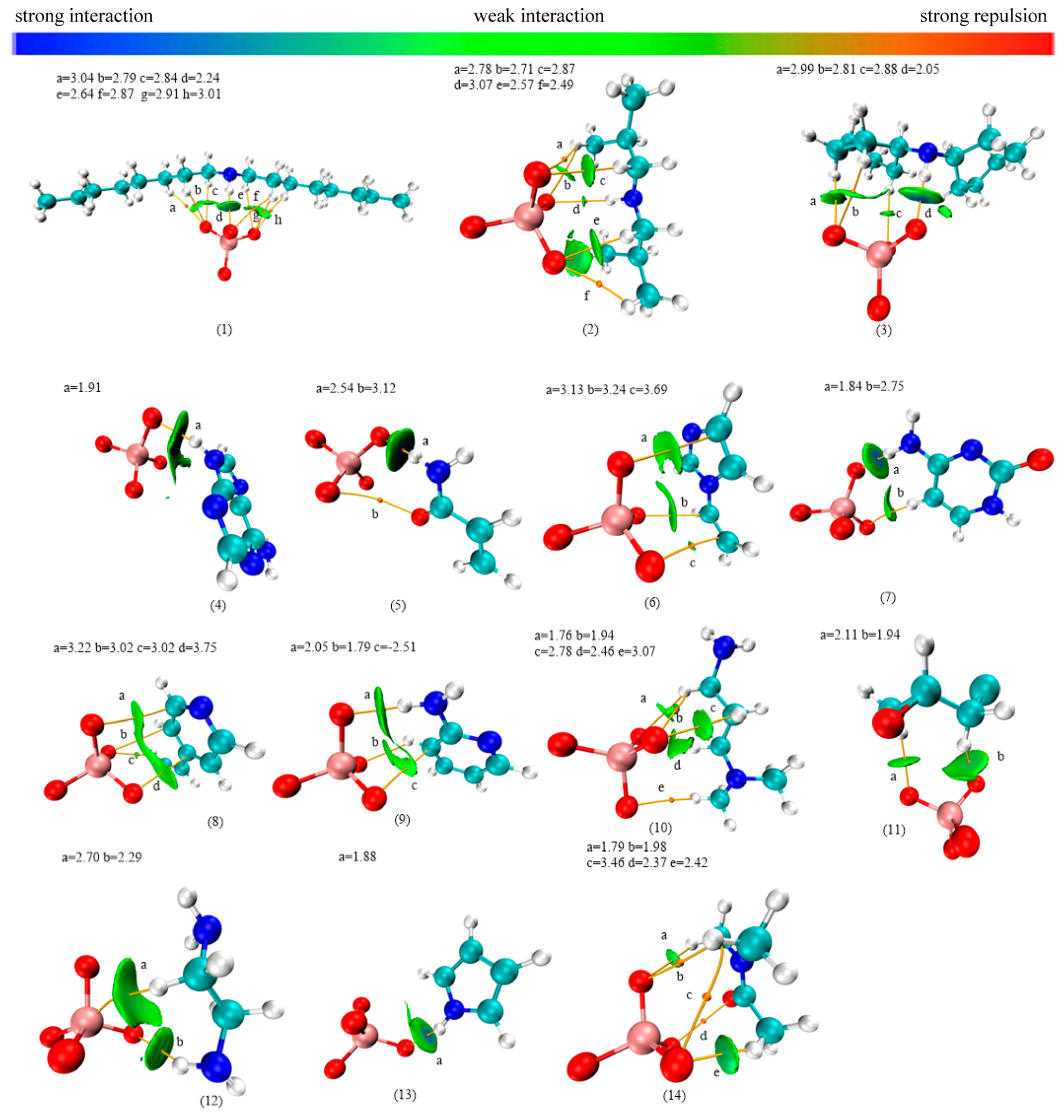
The geometries, geometric parameters, and IGMH gradient isosurfaces of the complexes between ReO_4_^−^ anion and 14 functional monomers. The numbers represent the distances of hydrogen bonds. Results are based on IGMH and AIM analyses, and the isosurfaces are generated with an isovalue of 0.005.

Figure 2 illustrates the most stable structures formed by ReO_4_^−^ interacting with the representative functional monomers. To quantitatively assess the adsorption capabilities of each functional monomer towards ReO_4_^−^, interaction energies (see Table 1) were further calculated based on the subtraction method as follows:

Δ*E* = *E*_tot_ − *E*_A_ − *E*_B_ (1)

Here, Δ*E* represents the interaction energy, *E*_tot_ denotes the total energy of the complex, *E_A_* and *E_B_* represent the electronic energies of the ReO_4_^−^ and monomer fragments, respectively, as they exist in their bound geometries within the intermolecular complex. This approach ensures that the calculated value reflects the pure electronic interaction energy rather than the overall binding energy which would otherwise include deformation penalties. According to this definition, a more negative ΔE value reflects a more energetically favorable interaction between the monomer and the anion. Analysis from Table 1 reveals that secondary amine functional monomers with symmetric structures (such as DOA, DIBA, DCHA) form more stable complexes compared to other types of monomers. This enhanced stability can be attributed to their longer alkyl chains, which not only provide a more extensive hydrophobic environment but also facilitate optimal spatial alignment for multiple hydrogen-bonding interactions, consistent with findings in amine extractant literature [34]. Following the aliphatic amines, nitrogen-containing heterocyclic monomers also exhibit significant adsorption characteristics. Specifically, cytosine (CY) forms the second most stable type of complex, followed by adenine (AD) in the third position. The strong performance of these ring-based monomers suggests that the presence of multiple nitrogen or oxygen sites can effectively compensate for the lack of long alkyl chains through localized electronic effects and synergistic coordination. Overall, while aliphatic amine functional monomers demonstrate the highest binding affinity towards ReO_4_^−^ among the monomer types tested, the heterocyclic structures CY and AD emerge as the next most competitive candidates due to their high density of functional groups and efficient spatial coordination with the perrhenate anion. Hydrogen bonding is a special class of attractive intermolecular forces that arise due to the dipole–dipole interaction between a hydrogen atom that is bonded to a highly electronegative atom and another highly electronegative atom which lies in the vicinity of the hydrogen atom. Typically, when the distance between the hydrogen atom and the nearby highly electronegative atoms is less than 3.5 Å [35], it is termed a hydrogen bond. In our work, two types of intermolecular hydrogen bonds were identified [36], i.e., N-H…O bonds and C-H…O bonds. From the perspective of distance, the hydrogen bond length of the former (ave.~2.10 Å in Figure 2) is shorter than that of the latter (ave.~2.80 Å in Figure 2). This geometric discrepancy reflects the fundamental difference in the proton-donating ability of the respective groups. The nitrogen atom, being more electronegative than carbon, significantly polarizes the N-H bond, resulting in a more positively charged hydrogen atom that can interact more strongly with the oxygen atoms of ReO_4_^−^. Consequently, the shorter N-H…O bonds represent the primary stabilizing force in the adsorption complexes, while the longer C-H…O bonds, although more numerous in some aliphatic chains, serve as secondary, auxiliary interactions. This indicates that the hydrogen bond interaction force of the former is greater than that of the latter, providing the essential directional stability required for effective anion capture.

**Figure 3:**
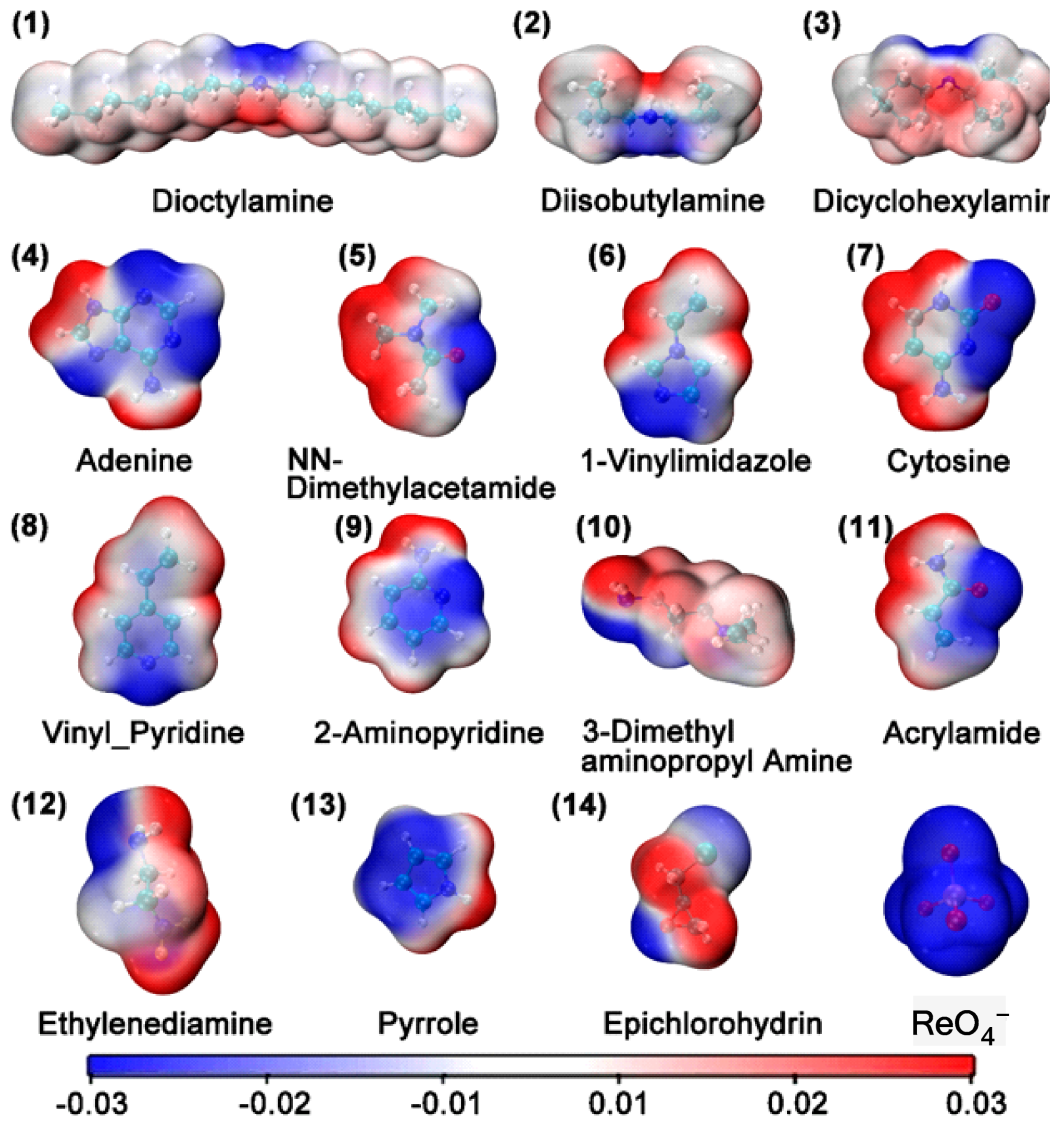
Molecular electrostatic potential (ESP) mapped on an electron density isosurface of 0.001 a.u. for the 14 functional monomers and ReO_4_^−^. The color scale ranges from −0.03 to 0.03 a.u. (red represents positive regions and blue represents negative regions).

It is well recognized that the overall interaction is determined not solely by hydrogen bond distances but by a composite of many effects including electronic distribution and steric configurations. Initially, we examined the average Re-O bond length in ReO_4_^−^, which was found to be 1.77 Å, consistent with previous reports (1.75~1.77 Å) [37], thereby confirming the reliability of our method and the chosen functional. Subsequently, we investigated the charge distribution using the Hirshfeld method [38] and the surface electrostatic potential of 14 functional monomers to identify the electronic origin of their binding affinities. Regarding charges, the oxygen atoms involved in hydrogen bonding interactions with ReO_4_^−^ indeed exhibit a partial loss of charge, albeit with minimal variations across different monomers, averaging around 0.01 e (see Table 1). This slight electronic redistribution suggests that while hydrogen bonding is the dominant force, it maintains a primarily non-covalent character with subtle polarization effects. Analysis of the surface electrostatic potential (ESP) has demonstrated its effectiveness in predicting active sites for adsorption studies [39,40]. In this study, owing to its strongly negative electrostatic potential (highlighted in blue in Figure 3), ReO_4_^−^ preferentially interacts strongly with the electrophilic regions that correspond to the positively charged regions within the functional monomers (highlighted in red in Figure 3) through hydrogen bonding (The ESP Figure of 14 complex ions are shown in Figure S2). This observation is perfectly confirmed in symmetric functional monomers like DOA, DIBA, and DCHA, where the convergence of high ESP values at the amine nitrogen consistently guides ReO_4_^−^ to adsorbs at the central positions. The high degree of spatial complementarity between the negative ESP of the oxo-anion and the positive ESP domains of the monomers underscores the electrostatic nature of the initial recognition process. 

To gain a more profound understanding of hydrogen bonding and its governing factors, we conducted a detailed analysis on the nature of N-H…O and C-H…O bonds within the top eight most stable complexes using energy decomposition analysis [41,42,43]. Unlike the conventional subtraction method, which provides only the net change in energy, energy decomposition analysis allows for the partition of the binding affinity into physically meaningful contributions. The total interaction energy (Δ*E_int_*) between fragments is decomposed into four distinct components:

Δ*E_int_* = Δ*E*_pauli_ + Δ*E*_elest_ + Δ*E*_orb_ + Δ*E*_disp_ (2)

Here, Δ*E_int_* represents the total interaction energy, Δ*E*_pauli_ accounts for Pauli repulsion arising from the exclusion principle between occupied orbitals, Δ*E*_elstat_ reflects the classical Coulomb interaction (ionic bonding), Δ*E*_orb_ denotes quasi-covalent bonding characterizing charge transfer and polarization, and Δ*E*_disp_ corresponds to long-range van der Waals interactions. Further emphasizing the local electronic structure details, the energy decomposition analysis results (see Table 2) highlight similarities and differences compared to previous subtractive interaction energies focused on overall energy differences (see Table 1). Specifically, while Table 1 ranks the monomers by their total thermodynamic stability, the energy decomposition analysis results provide a deeper insight into the competitive balance between attractive forces and Pauli repulsion, revealing why certain monomers with similar total energies exhibit distinct adsorption behaviors.

In particular, the amine types consistently exhibit strong adsorption capabilities towards ReO_4_^−^. The energy decomposition analysis results in Table 2 demonstrate that the electrostatic term Δ*E*_elstat_ (~37.97%), the orbital term Δ*E*_orb_ (~34.41%), and the dispersion term Δ*E*_dis_ (~27.62%) collectively contribute significantly to the total interaction energy Δ*E_int_*. This tripartite dominance underscores their critical roles in mediating the interactions between ReO_4_^−^ and each functional monomer, suggesting a balanced interplay where coulombic attraction, covalent character (charge transfer), and van der Waals forces are equally indispensable. Conversely, aromatic and π-conjugated systems in AD and CY functional monomers exhibit stronger adsorption towards ReO_4_^−^ (>15 kcal/mol) compared to the amines (<15 kcal/mol). Examining the distribution of attractive contributions reveals that the electrostatic term Δ*E*_elstat_ constitutes ~55.33%, the orbital term Δ*E*_orb_ constitutes ~34.08%, and the dispersion term Δ*E*_disp_ constitutes ~10.59%. Notably, the electrostatic term in these heterocyclic systems predominates over the dispersion term, highlighting its compressive effect on the intermolecular distance. This shift in energy distribution indicates that the rigid planar structure of AD and CY, coupled with their multiple N-H hydrogen bond donors, enhances the local electric field intensity. Consequently, the electrostatic interaction emerges as the primary driving force, effectively promoting a closer spatial approach of the anion toward the functional sites through enhanced Coulombic attraction. Simultaneously, the relatively diminished percentage of the dispersion term reflects a more localized and directional binding mode, contrasting with the non-specific, long-range van der Waals stabilization observed in the long-chain amine counterparts.

Furthermore, a comparative analysis of the interaction energies across the full suite of studied anions reveals that these integrated structural and electronic features confer a distinct thermodynamic preference for ReO_4_^−^ over competing species such as Cl^−^ and SO_4_^2−^. Taking the CY^−^ based complex as a representative case, the interaction energy with ReO_4_^−^ is markedly more negative than those involving the other anions, highlighting the superior capture potential of this specific monomer architecture. This pronounced selectivity is fundamentally rooted in the larger ionic radius and higher polarizability of ReO_4_^−^, which promotes enhanced dispersion-driven interactions and facilitates the formation of robust multi-site hydrogen bonding networks. Unlike smaller or more highly hydrated anions like Cl^−^ or SO_4_^2−^, which often incur significant energetic penalties during the desolvation process due to their high charge density, the relatively low hydration energy of the soft ReO_4_^−^ anion allows it to approach the functional sites more effectively.

To provide a spatial manifestation of these energetic findings and identify interaction regions in real molecular space, independent gradient model based on Hirshfeld partition (IGMH) and atoms in molecules (AIM) analyses were performed (see Figure 2). For AD and CY functional monomers, the isosurfaces provide a visual confirmation of their complex binding modes, where both strong hydrogen bonding interactions (evident from shorter N-H…O bond lengths) and anion-heterocycle interactions between ReO_4_^−^ and aromatic rings (evident from green regions between ReO_4_^−^ and aromatic rings) are observed. These features indicate that the adsorption process involves the simultaneous contribution of diverse non-covalent interactions. Therefore, summarizing the findings, while amine-based systems generally exhibit greater overall stability in ReO_4_^−^ adsorption according to the calculated interaction energies, aromatic systems with nitrogen-containing rings leverage a combination of degenerate hydrogen bonding and anion-heterocycle interactions at the local electronic structure level, offering superior performance in certain contexts. This comprehensive analysis of the local electronic environment elucidates how these specific functional groups enhance the affinity toward anions beyond simple electrostatic attraction.

In addition, we extended our investigation to include other anions such as Cl^−^, F^−^, and SO_4_^2−^ to assess the universality and selectivity of these functional monomers. Our analysis revealed that Cl^−^ and SO_4_^2−^ exhibit similar adsorption capabilities towards the 8 functional monomers (see Table S3) and share comparable adsorption sites (detailed in Figures S3 and S4) with ReO_4_^−^, underscoring the dominant role of classical hydrogen bonding interactions in stabilizing these anion-monomer complexes. However, significant distinctions emerged with F^−^, which exhibits a much higher charge density, stronger electron withdrawing capabilities and lesser steric repulsion owing to its smaller ionic radius. Specifically, F^−^ displayed notably different interaction profiles with AD and CY functional monomers compared to Cl^−^, SO_4_^2−^ and ReO_4_^−^. The total interaction energies with AD and CY were calculated at −90.44 and −79.15 kcal/mol (see Table S3), respectively, which are significantly higher than those observed with the other three anions. This remarkable energy divergence is primarily attributed to F^−^ abstracting a proton from the amino or imino groups of AD and CY, leading to the disruption of the N-H bonds on these monomers (see Figure S5). This transformation signifies a transition from non-covalent Physisorption to a Chemisorption-like process, which is akin to a chemical shift from weak N-H…F hydrogen bonding to stronger N…H-F interactions. The significant structural deformation of the monomers, characterized by the elongation and eventual cleavage of N-H bonds, along with the substantial energy release (exceeding 70 kcal/mol), further confirm that F^−^ interacts with these nitrogen-containing rings through a distinct deprotonation mechanism rather than simple surface coordination.

## 3. Materials and Methods

The computational strategy in this study followed a hierarchical approach designed to balance computational efficiency with high-level chemical precision, ensuring a thorough exploration of the complex potential energy surface (PES). Initially, we explored adsorption structures by randomly generating and screening interactions between ReO_4_^−^ and 14 different functional molecules. For each molecule, ten random adsorption configurations were considered at different positions and orientations, resulting in a total of 140 initial adsorption structure hypotheses [44]. This stochastic sampling was crucial to avoid local energy minima and identify the most probable binding orientations.

Geometry optimization for all species was conducted using DFT calculations with the third-generation dispersion correction implemented in the Gaussian 09 package [45]. Initial structural modeling and screening utilized the M06-D3 functional with the LANL2DZ basis set for the Re atom and the 6-31G(d) basis set for other atoms. Structures were ranked based on energy, and the three lowest-energy configurations from each adsorption complex were selected for subsequent refinement, totaling 42 distinct adsorption complexes (M06-D3/RECP/6-311G(d,p)). Subsequently, we rigorously confirmed the optimal adsorption sites among these 42 identified structures using high-precision computational methods. The meta-hybrid GGA functional M06 [46,47,48], known for its robust performance in studying electronic structure properties across various chemical families, organometallic compounds, and non-covalent interactions, was utilized for all calculations. The lowest-energy configurations from each adsorption complex were selected, resulting in the identification of 14 adsorption complexes with optimal adsorption sites (see Table S2). The consistency between the initial screening and final high-precision refinement reinforces the robustness of our structural predictions. For the comparative analysis of other investigated anions, the adsorption complexes were evaluated using the same M06-D3/RECP/6-311G(d,p) level of theory, and the corresponding results are summarized in Table S3. Vibrational frequencies were calculated at the same level to ensure energy minimization on the potential energy surface [49,50,51,52]. The Cartesian coordinates of all optimized geometries reported in this study are provided in the Supporting Information (see cartesian coordinates at supporting information).

To accurately describe the anionic nature of ReO_4_^−^ and the delocalized electron density in its complexes, we performed additional single-point energy calculations using a larger basis set containing diffuse functions. Based on the optimized geometries, final interaction energies were refined using the M06-D3/RECP/6-311+G(d,p) level of theory. This addition of diffuse functions is essential for mitigating Basis Set Superposition Error (BSSE) and providing a precise description of anionic interaction energies. To simulate realistic industrial and environmental conditions, the Solvation Model based on Density (SMD) with water as the solvent was employed for all calculation stages (optimizations and single-point energies). This accounts for the hydration effects that compete with monomer binding in aqueous solutions. All calculations for the ReO_4_^−^ complexes and the other investigated anions utilized a consistent computational protocol involving the same functional, basis sets, and solvent models to guarantee reproducibility. Electronic structure analysis of the M06-D3/RECP/6-311G(d,p) optimized structures was conducted using Multiwfn package [53] and Amsterdam Density Functional (ADF) program [54]. In the Multiwfn package, the independent gradient model based on Hirshfeld partition (IGMH) and atoms in molecules (AIM) analyses [55,56] was performed to explore interactions between the adsorbed monomer and ReO_4_^−^. ADF calculation employed the Perdew–Burke–Ernzerhof (PBE) [57] functional with empirical dispersion corrections [58], considering scalar relativistic (SR) effects within the zeroth-order regular approximation (ZORA) [59]. A triple-ζ double polarization (TZ2P) [60] basis set among uncontracted Slater-type orbital (STO) basis sets was used with a small frozen core. Energy decomposition analysis with natural orbital for chemical valence (NOCV) [61], was employed to explore the nature of interactions between ReO_4_^−^ and monomers. It should be noted that while the M06-D3 functional was used for the initial energetic screening and geometry optimization, the choice of the PBE-D3/TZ2P level for EDA is based on its robust implementation of the Morokuma-Ziegler scheme and the superior performance of Slater-type orbitals in describing electronic partitioning. Although minor variations in the relative stability rankings of the complexes (such as for Cytosine and Adenine) may arise due to the different treatment of exchange-correlation effects between GGA and meta-hybrid functionals, the qualitative insights into the nature of the intermolecular forces remain consistent and provide a comprehensive physical picture of the adsorption process.

Furthermore, the Beijing Density Functional (BDF) program package [62] calculations were performed to investigate the relativistic electronic structure of ReO_4_^−^. The B3LYP-D3 functional was employed. Scalar relativistic (SR) effects were treated using cc-pVTZ-PP (Re) and aug-cc-pVTZ (O) basis sets with the COSMO solvation model. For spin-orbit coupling (SOC) analysis, the exact two-component (X2C) relativistic Hamiltonian was used with cc-pVTZ-DK (Re) and aug-cc-pVTZ-DK (O) basis sets. SOC effects were evaluated via state interaction of the lowest six singlet and six triplet excited states from TD-DFT calculations [63].

## 4. Conclusions

In this study, we employed density functional theory (DFT) to investigate the adsorption behaviors of ReO_4_^−^, Cl^−^, SO_4_^2−^, and F^−^ anions onto several distinct functional monomers. Our computational approach involved multilevel optimizations to explore both the geometric configurations and energetic characteristics of the complexes formed. We systematically analyzed the interactions, focusing on hydrogen bonding and anion-heterocycle interactions, to discern the underlying mechanisms governing anion adsorption. Our findings underscored the selective affinity of ReO_4_^−^, Cl^−^, and SO_4_^2−^ towards amine-type functional monomers, which are adept at forming stable hydrogen bonds. Conversely, fluorine anions (F^−^) exhibited unique interactions with adenine (AD) and cytosine (CY), involving significant structural alterations. These insights highlight the pivotal role of molecular structure and electronic properties in dictating the adsorption efficiency of functional materials. Our study advances the understanding of anion adsorption mechanisms on functional monomers, revealing diverse interaction patterns that can be harnessed for designing selective adsorbents. By integrating computational insights with experimental validations, future research can leverage these findings to develop tailored materials for environmental remediation and industrial separations, thereby addressing critical challenges in water purification and chemical processing.

## Figures and Tables

**Table 1:** Interaction energy between ReO_4_^−^ and 14 functional monomers, and the Hirshfeld charge distribution analysis of oxygen atoms on ReO_4_^−^.

Number	Complexes	Δ*E* (kcal/mol)	Charge			
			O_1_	O_2_	O_3_	O_4_
1	DOA_ReO_4_^−^	−12.11	−0.464	−0.471	−0.457	−0.499
2	DIBA_ReO_4_^−^	−10.43	−0.484	−0.450	−0.468	−0.500
3	DCHA_ReO_4_^−^	−10.76	−0.473	−0.430	−0.489	−0.498
4	AD_ReO_4_^−^	−9.83	−0.450	−0.484	−0.488	−0.490
5	DMAC_ReO_4_^−^	−9.03	−0.455	−0.462	−0.493	−0.502
6	VIM_ReO_4_^−^	−9.38	−0.495	−0.487	−0.484	−0.499
7	CY_ReO_4_^−^	−10.01	−0.431	−0.488	−0.484	−0.488
8	4-VP_ReO_4_^−^	−9.38	−0.493	−0.486	−0.485	−0.500
9	2-AP_ReO_4_^−^	−8.02	−0.459	−0.474	−0.483	−0.493
10	DMAPA_ReO_4_^−^	−7.85	−0.462	−0.477	−0.495	−0.502
11	AM_ReO_4_^−^	−8.16	−0.437	−0.494	−0.493	−0.491
12	EDA_ReO_4_^−^	−8.64	−0.489	−0.487	−0.461	−0.500
13	PY_ReO_4_^−^	−8.91	−0.440	−0.489	−0.490	−0.491
14	ECH_ReO_4_^−^	−7.75	−0.474	−0.477	−0.499	−0.493

**Table 2:** Energy Decomposition Analysis results for the different forms of interacting fragments at the PBE-D3/TZ2P Level of theory, taking all the fragments in the singlet state as interacting fragments at the corresponding frozen complexes. Energy values are given in kcal/mol.

Number	Complexes	^a^ Δ*E*_pauli_	^a^ Δ*E*_elest_	^a^ Δ*E*_orb_	^a^ Δ*E*_disp_	Δ*E_int_*
1	DOA vs._ReO_4_^−^	8.65	−8.62 (37.87%)	−8.03 (35.28%)	−6.11 (26.85%)	−14.11
2	DIBA vs. ReO_4_^−^	7.02	−7.47 (38.17%)	−6.19 (31.63%)	−5.91 (30.20%)	−12.55
3	DCHA vs. ReO_4_^−^	9.65	−9.23 (40.39%)	−8.27 (36.19%)	−5.35 (23.41%)	−13.2
4	AD vs. ReO_4_^−^	12.6	−14.40 (51.97%)	−10.01 (36.12%)	−3.30 (11.91%)	−15.1
5	DMAC vs. ReO_4_^−^	7.92	−5.73 (35.46%)	−5.58 (34.53%)	−4.85 (30.01%)	−8.24
6	VIM vs. ReO_4_^−^	3.51	−1.30 (14.93%)	−3.15 (36.17%)	−4.26 (48.91%)	−5.21
7	CY vs. ReO_4_^−^	13.5	−20.16 (58.69%)	−11.01 (32.05%)	−3.18 (9.26%)	−20.85
8	4-VP vs. ReO_4_^−^	4.63	−1.96 (19.35%)	−3.43 (33.86%)	−4.74 (46.79%)	−5.50

^a^ Within this energy decomposition scheme the attractive and repulsive terms are negative and positive.

## Data Availability

The original contributions presented in this study are included in the article/Supplementary Material. Further inquiries can be directed to the corresponding author.

## Supplementary Materials

The following supporting information can be downloaded at: https://www.mdpi.com/article/10.3390/ijms27093881/s1.
