# Supplementary material for: Theoretical Investigation on the Selective Adsorption of ReO4− by Functional Monomers: The Role of Hydrogen Bonding and Anion–Heterocycle Interactions
Source: Int J Mol Sci. 2026 Apr 27;27(9):3881. doi: 10.3390/ijms27093881 (PMC13164512; doi:10.3390/ijms27093881)
Supplement: Supplementary file 1 [file ijms-27-03881-s001.zip › ijms-4223949-supplementary.pdf]

Supporting Information

Theoretical Investigation on the Selective Adsorption of ReO_4^- by Functional Monomers: The Role of Hydrogen Bonding and Anion-heterocycle Interactions

Jiongyao Wu[1], Bo Wang[2], Yang Gao[3,*]

1. Department of Physics, University of Duisburg-Essen, Lotharstrasse 1, D-47057 Duisburg, Germany

2. School of Science, Northeast Electric Power University, Jilin 131200, China

3. Institute of Fundamental and Frontier Sciences, University of Electronic Science and Technology of China, Chengdu, Sichuan 611731, China

*E-mail : ygaoxs@gmail.com

The exploration of the potential energy surface (PES) was facilitated by the molclus program utilizing its automated stochastic search and clustering algorithms. During the initial generation phase, the genmer module of molclus was employed to create random structural guesses for each monomer-anion complex. The ReO_4^- anion was randomly positioned within a defined spatial range around the functional monomer, and the program automatically filtered out any structures containing atomic overlaps or excessively large intermolecular distances. For each monomer system, a total of 140 initial structural hypotheses were generated and subsequently screened based on energy using the molclus toolkit. A crucial aspect of this process was structural clustering, where redundant or highly similar configurations were removed by comparing total electronic energies and Root-Mean-Square Deviations (RMSD). This rigorous redundancy check ensures that the remaining ten structures for each monomer represent a diverse and representative set of local minima across the potential energy surface. Finally, following a hierarchical computational strategy, the three lowest-energy configurations from each set were selected for high-level refinement at the M06-D3/RECP/6-311G(d,p) level of theory. This approach ensures that the most energetically favorable binding motifs identified during the stochastic sampling are accurately characterized and described with high chemical precision.

Table S1. Relative energies (kcal/mol) for complexes formed ReO_4^- and 14 functional monomers. Calculations were performed using M06-D3/LANL2DZ-Re /6-31G* levels.

Complexes	1	2	3	4	5	6	7	8	9	10
DOA_ReO_4^-	0.000	3.291	3.602	5.657	6.283	7.406	9.196	9.922	9.985	10.091
DIBA_ReO_4^-	0.000	1.571	3.285	8.329	8.578	8.971	8.986	9.855	10.336	12.132
DCHA_ ReO_4^-	0.000	1.081	1.237	1.306	2.042	2.072	3.494	5.038	5.182	6.201
AD_ ReO_4^-	0.000	0.115	2.116	2.916	5.253	5.531	7.914	7.934	8.188	9.268
DMAC_ ReO_4^-	0.000	0.976	3.062	3.277	5.575	6.017	6.181	6.296	8.704	10.009
VIM_ ReO_4^-	0.000	0.446	1.167	1.743	1.816	2.555	2.749	3.343	3.759	5.526
CY_ ReO_4^-	0.000	1.178	1.382	1.610	2.079	2.436	2.772	5.487	7.487	8.586
4-VP_ ReO_4^-	0.000	1.168	1.257	1.297	1.397	2.363	2.385	3.335	3.795	6.155
2-AP_ ReO_4^-	0.000	0.746	0.989	2.082	2.460	4.332	5.111	7.344	7.446	8.240
DMAPA_ ReO_4^-	0.000	0.430	2.784	3.294	3.673	3.723	3.752	3.973	5.121	6.767
AM_ ReO_4^-	0.000	2.464	2.823	2.837	3.009	4.843	7.337	10.180	10.498	11.102
EDA_ ReO_4^-	0.000	2.589	2.871	3.555	3.915	3.960	4.132	4.144	5.686	5.726
PY_ ReO_4^-	0.000	1.861	6.321	7.379	8.705	8.772	9.158	9.556	9.751	11.278
ECH_ ReO_4^-	0.000	0.262	1.892	2.206	2.592	4.903	5.139	6.524	10.424	10.675

Table S2. Relative energies (kcal/mol) for complexes formed ReO_4^- and 14 functional monomers. Calculations were performed using M06-D3/RECP-Re/6-311G(d,p) levels.

Complexes	1	2	3
DOA_ReO_4^-	0.000	3.020	3.170
DIBA_ReO_4^-	0.000	0.212	2.186
DCHA_ ReO_4^-	0.000	0.561	1.039
AD_ ReO_4^-	0.000	1.047	238.649
DMAC_ ReO_4^-	0.000	0.155	0.588
VIM_ ReO_4^-	0.000	0.900	2.967
CY_ ReO_4^-	0.000	0.181	0.425

4-VP_ ReO_4^-	0.000	0.095	2.742
2-AP_ ReO_4^-	0.000	0.015	1.549
DMAPA_ ReO_4^-	0.000	1.700	1.770
AM_ ReO_4^-	0.000	0.466	0.689
EDA_ ReO_4^-	0.000	0.244	0.463
PY_ ReO_4^-	0.000	0.201	2.883
ECH_ReO_4^-	0.000	0.774	0.844

Table S3. Interaction energy between Anions and 8 functional monomers. Calculations were performed using M06-D3/RECP-Re/6-311G(d,p) levels.

Number	Complexes	ReO_4^-	Cl^-	F^-	SO_4^{2-}
1	DOA_Anion	-10.92	-5.33	-20.93	-12.02
2	DIBA_Anion	-9.18	-4.97	-22.81	-11.59
3	DCHA_Anion	-8.84	-5.22	-22.53	-12.03
4	AD_ Anion	-8.00	-6.36	-90.44	-7.86
5	DMAC_ Anion	-7.60	-3.34	-16.63	-6.94
6	VIM_ Anion	-7.45	-1.22	-19.31	-6.98
7	CY_ Anion	-7.39	-6.10	-79.15	-10.45
8	4-VP_ Anion	-6.92	-2.51	-17.56	-5.98

To provide a foundational electronic basis for the adsorption studies, we first analyzed the molecular orbital (MO) energy levels of ReO_4^- by comparing scalar relativistic (SR) and spin-orbit coupling (SOC) effects. The transition from SR to SOC leads to a discernible lifting of orbital degeneracy, characterized by energy level splitting. Despite this relativistic fine-tuning, the overall electronic stability remains robust, as evidenced by the negligible fluctuation in the ΔE_{H-L} gap, which shifts only slightly from -6.42 eV to -6.36 eV (a difference of only 0.06 eV). This wide and stable energy gap confirms that the fundamental electronic structure of ReO_4^- is well-preserved under relativistic perturbations（**Figure S1**）.

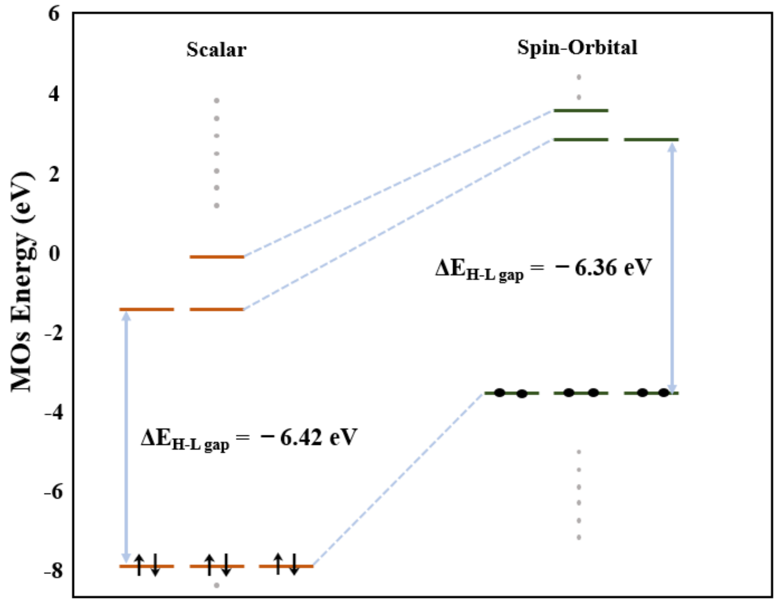

Figure S1. MO energy level diagrams of ReO_4^- under Scalar and Spin-Orbital relativistic effects.

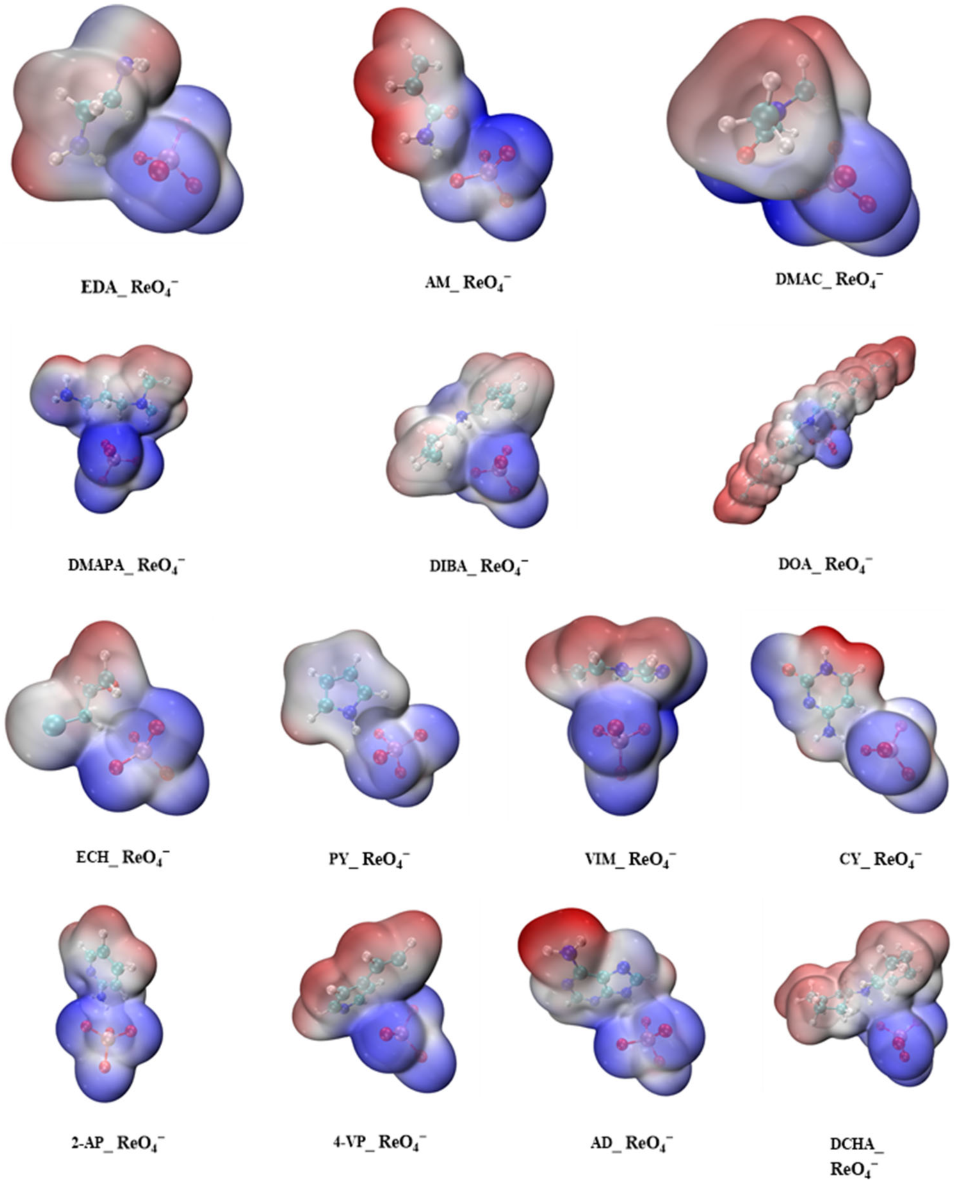

Figure S2. Molecular electrostatic potential (ESP) mapped on an electron density isosurface of 0.001 a.u. for the 14 complexes. The color scale ranges from −0.22 to 0.00 a.u.

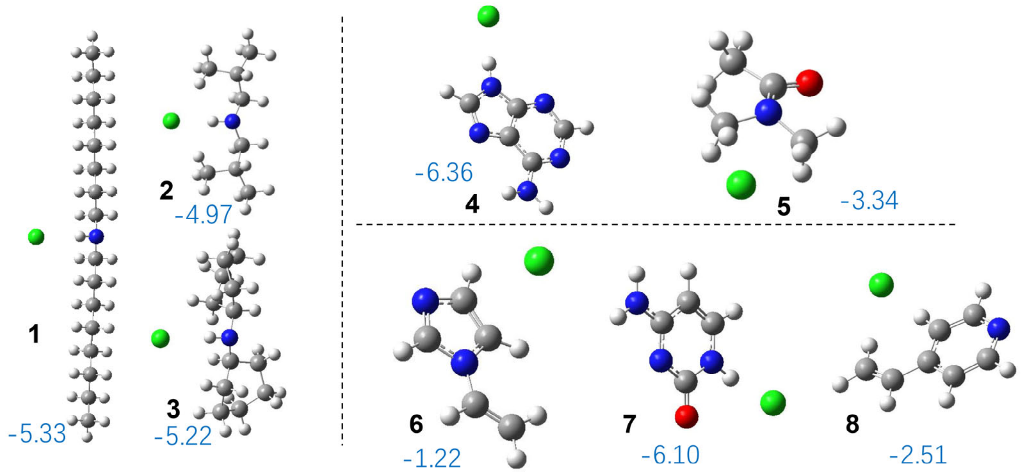

Figure S3. Geometries of the complexes between Cl^- anion and 8 functional monomers.

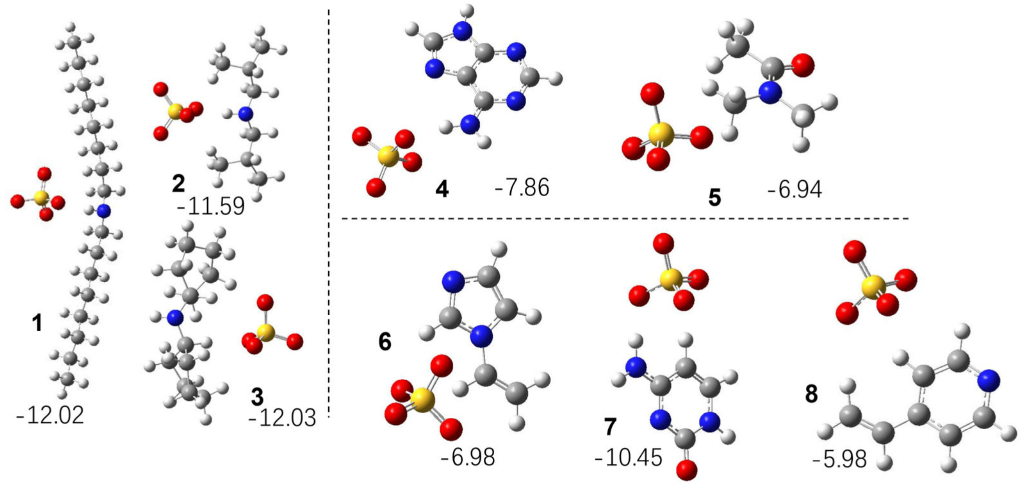

Figure S4. Geometries of the complexes between SO_4^{2-} anion and 8 functional monomers.

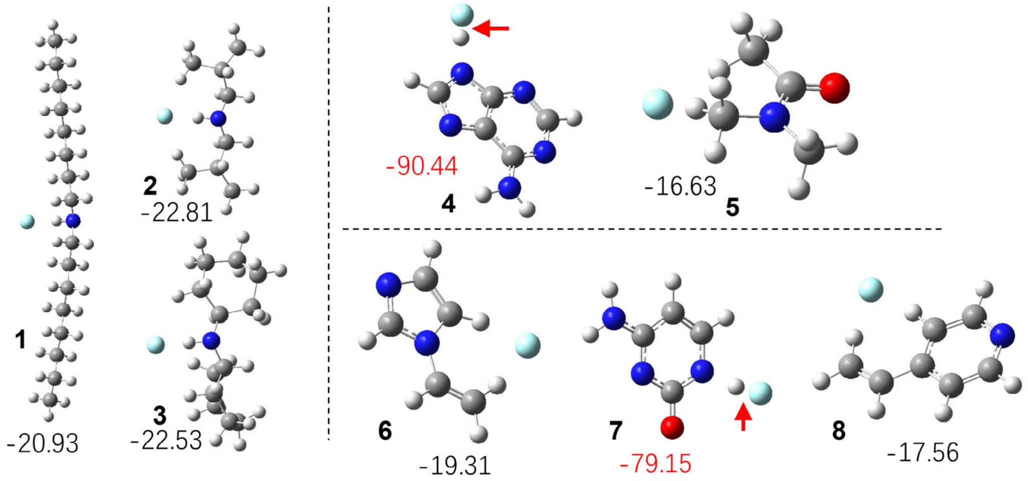

Figure S5. Geometries of the complexes between F^- anion and 8 functional monomers.

Cartesian coordinates

M06-D3/RECP-Re/6-311+G(d,p) levels level with SMD (Solvent: Water)

Ethylenediamine

C	-0.80502900	0.89775600	0.36809500
H	-0.11572700	0.58072900	-0.42948300
H	-1.73911400	1.18973500	-0.12718600
C	-1.06214400	-0.28840300	1.26641200
H	-0.18752400	-0.41648800	1.92922700
H	-1.91491700	-0.07421000	1.92136900
N	-0.30285100	2.03011300	1.14302800
H	0.62557200	1.78291000	1.48244700
H	-0.15414500	2.81406000	0.51311300
N	-1.35165600	-1.47553100	0.46574700
H	-0.47560700	-1.75358600	0.02077400
H	-1.59117200	-2.24036300	1.09037800

Acrylamide

C	-0.45611200	-0.30896400	0.73701000
C	-1.43448100	-0.91687300	1.67275900
H	-2.20492600	-0.26488100	2.07972200
N	-0.67282100	0.98217300	0.45572500
H	-1.43203000	1.48274900	0.89402100
H	-0.00986600	1.48232400	-0.14353800
C	-1.37605600	-2.19925300	2.00134100
H	-2.08990400	-2.64426200	2.68640400
H	-0.60590200	-2.84608700	1.58968500
O	0.48447400	-0.94738300	0.25349700

NN-Dimethylacetamide

N	0.36644800	1.17207900	0.14063000
C	1.21559100	0.31076600	-0.47034900
O	1.40740600	0.36540800	-1.68859100
C	-0.18186300	0.96350800	1.46811400
H	-0.23277000	1.91720100	2.00211400
H	-1.20019600	0.55722100	1.40118200
H	0.42906700	0.27919200	2.05342600
C	-0.38664200	2.09035200	-0.69493500
H	-0.80097200	2.87856600	-0.06264800
H	0.26345600	2.54458500	-1.44325900
H	-1.21197000	1.57974500	-1.20721300
C	1.93800700	-0.69515000	0.37658200
H	1.24262600	-1.47284800	0.71159800
H	2.71680300	-1.15567600	-0.23187900
H	2.39108600	-0.24289600	1.26287000

3-Dimethylaminopropylamine

N	-1.09350500	-0.17153400	-1.26862200
C	-1.54140200	-1.47989500	-1.71505400
H	-1.74604800	-2.13838200	-0.86705400
H	-2.46767400	-1.38060100	-2.28822000
H	-0.79015300	-1.97369000	-2.36176100
C	-0.94091100	0.69258300	-2.42729700
H	-1.89846400	0.80623500	-2.94403800
H	-0.60131200	1.68553900	-2.11509600
H	-0.20671400	0.29185200	-3.15222300
C	0.20336400	-0.28126600	-0.60704600
H	0.53553900	0.73578200	-0.36173900
H	0.95458900	-0.69198400	-1.31548200
C	0.20212800	-1.11512900	0.65712900
H	0.10949500	-2.18411400	0.41746700
H	-0.66385000	-0.85111300	1.27852100
C	1.47116900	-0.90255700	1.45474800
H	1.52586300	0.14600800	1.77142200
H	2.33959000	-1.06962000	0.79389000
N	1.48285900	-1.74393700	2.64886000
H	1.50333200	-2.71640300	2.34726500
H	2.36263200	-1.59980300	3.13808800

Diisobutylamine

N	0.08603200	-0.94451500	-0.46416300
H	-0.76913200	-0.55263900	-0.06892600
C	1.07209300	-0.98312800	0.60802000
H	2.01781000	-1.34967800	0.18439100
H	1.28490300	0.03262000	0.99530800
C	0.67933700	-1.87344900	1.77506300
H	0.63426400	-2.90970400	1.40580300
C	0.52927400	-0.03621400	-1.51506800
H	1.48536400	-0.41264700	-1.90521200
H	0.74146100	0.97470500	-1.11005600
C	-0.45136800	0.10808900	-2.66633800
H	-0.60399900	-0.88747100	-3.11087800
C	-0.68029500	-1.51008000	2.35565500
H	-1.49833800	-1.66501000	1.64419300
H	-0.89920700	-2.12322900	3.23707000
H	-0.69686800	-0.45820900	2.66854400
C	1.74990600	-1.77816500	2.85102600
H	1.54879200	-2.46311900	3.68154100
H	2.74525900	-2.00957600	2.45476000
H	1.78430800	-0.76019900	3.26158900
C	-1.79555300	0.65058500	-2.20593200

H	-2.46576700	0.80383900	-3.05902000
H	-2.30736700	-0.02133600	-1.50855200
H	-1.66180100	1.61973900	-1.70519400
C	0.14631100	1.02484700	-3.72061100
H	1.11951000	0.66200400	-4.07045600
H	-0.51248700	1.11381400	-4.59099100
H	0.29247000	2.03343500	-3.31016400

Dioctylamine

N	0.54497700	0.67651600	-0.68642700
H	0.85201900	0.29393900	0.21203400
C	0.13453200	-0.45894500	-1.50172400
H	-0.22073300	-0.08637300	-2.47371200
H	-0.72863500	-0.98537300	-1.04670400
C	1.26119500	-1.44406700	-1.71309200
H	2.07998000	-0.95286300	-2.25837700
H	1.67054000	-1.73542400	-0.73295600
C	0.82406300	-2.68842300	-2.46198900
H	0.37233800	-2.39883300	-3.42355100
H	0.03072400	-3.19614400	-1.89230600
C	1.96533200	-3.65706600	-2.71017700
H	2.76480100	-3.14232700	-3.26564000
H	2.40828400	-3.94922300	-1.74515700
C	1.54925500	-4.90198800	-3.47086300
H	1.09204200	-4.60806900	-4.42860100
H	0.76152800	-5.42774900	-2.90907100
C	2.70194000	-5.85311700	-3.73557400
H	3.48752600	-5.32555600	-4.29922500
H	3.16303000	-6.14436800	-2.77876600
C	2.29442500	-7.10235900	-4.49579600
H	1.82951900	-6.80999900	-5.44835000
H	1.51441300	-7.63089500	-3.92911700
C	3.46286500	-8.03299300	-4.75733700
H	3.15745400	-8.93513300	-5.29684200
H	4.23610800	-7.53542400	-5.35490700
H	3.93079400	-8.35121400	-3.81804100
C	-0.58524600	1.55099100	-0.40731000
H	-0.88611600	2.05410300	-1.33775700
H	-1.46648800	0.96666200	-0.07855300
C	-0.26118900	2.57547900	0.65850700
H	0.25447800	2.06959600	1.49024900
H	0.44733600	3.31810800	0.26426700
C	-1.49988200	3.26512100	1.19874700
H	-2.06405000	3.72271100	0.37137000
H	-2.16466100	2.50407800	1.63563800

C	-1.18970300	4.31551800	2.24888800
H	-0.54807000	3.86979700	3.02506100
H	-0.59860900	5.12668800	1.79674600
C	-2.43088300	4.89328500	2.90352200
H	-3.08687200	5.32605600	2.13213900
H	-3.00668000	4.07628800	3.36564800
C	-2.12199800	5.94499500	3.95296300
H	-1.43539200	5.52049500	4.70234700
H	-1.57752700	6.78051400	3.48577800
C	-3.35755100	6.48015200	4.65504400
H	-4.04777700	6.89521800	3.90659400
H	-3.89233900	5.64400500	5.12810800
C	-3.02772600	7.53410400	5.69418000
H	-2.52304700	8.39369200	5.23699000
H	-3.92302200	7.90688700	6.20161300
H	-2.35532500	7.13161200	6.46149000

Epichlorohydrin

C	0.09794500	-1.43943200	-1.14447900
C	1.12838000	-0.49470000	-1.54497400
H	-0.03018300	-2.35879400	-1.71514700
H	1.75826300	-0.71944100	-2.40212300
H	0.99893600	0.55925400	-1.30143000
O	1.31441900	-1.34857700	-0.41823700
C	-1.09327800	-0.95108300	-0.39353000
H	-1.51500300	-1.70971700	0.26715600
H	-0.85812500	-0.04586600	0.17036700
Cl	-2.40269300	-0.50457500	-1.56716800

Pyrrole

C	0.11197300	1.07510400	0.51328100
C	-0.67252000	1.67961000	1.46561300
C	-1.09963500	0.66315400	2.36074200
C	-0.55711900	-0.52038500	1.91988100
N	0.18059800	-0.25807200	0.80150700
H	0.67766300	-0.92939900	0.21137600
H	0.63274800	1.48249600	-0.34225800
H	-0.91088800	2.73323900	1.51651200
H	-1.73236800	0.78572700	3.22927400
H	-0.64029700	-1.52437700	2.31006200

1-Vinylimidazole

C	-0.03345300	-1.81127100	0.50470100
C	1.05149600	-0.82834200	2.04920600
C	0.06560800	0.03667000	1.68930900

N	-0.64408600	-0.60672900	0.69512900
H	-0.39994700	-2.51172600	-0.23431000
H	1.80891200	-0.68447300	2.80640900
H	-0.20283100	1.01796600	2.04849700
N	0.98491400	-1.98861400	1.30608600
C	-1.73078000	-0.13823100	-0.06187400
H	-2.13620400	-0.89887000	-0.72259800
C	-2.22754800	1.08748200	0.00199000
H	-3.07757400	1.34909200	-0.61620900
H	-1.81772900	1.85419500	0.65142200

Cytosine

C	-0.27429600	1.93828200	-0.19029900
C	0.13301400	0.66153700	-0.04472000
C	-0.02381600	-0.19299400	-1.18132100
C	-0.92071500	1.51245400	-2.47527300
H	-0.21569200	2.67742700	0.60007300
H	0.55470900	0.29717000	0.88463400
N	-0.79210600	2.35497100	-1.37018100
H	-1.09823300	3.31366100	-1.48628100
N	-0.52633900	0.22745700	-2.33974200
O	-1.38828100	1.97808600	-3.51617900
N	0.34422000	-1.47267400	-1.08246200
H	0.75696300	-1.84037500	-0.22197500
H	0.24160700	-2.06742200	-1.89176400

2-Aminopyridine

C	-1.99029400	0.46151200	-1.88593400
C	-1.08381700	-0.37728500	-2.50066800
C	-0.17886400	-1.05521600	-1.68262300
C	-0.22555900	-0.86642100	-0.32156500
C	-1.17375400	0.02708900	0.20624800
H	-2.71770400	1.00505600	-2.48693800
H	-1.08092700	-0.49733200	-3.57797500
H	0.46470100	-1.37097200	0.34760100
N	-2.05009100	0.67805600	-0.56689100
H	0.55835000	-1.72811000	-2.11144900
N	-1.25399300	0.22549500	1.55950100
H	-0.39582700	0.08169000	2.09052700
H	-1.77494900	1.04992400	1.82775600

4-Vinylpyridine

C	1.13500800	0.02770100	-1.72365900
C	-0.15066200	-0.13361500	-1.24534300
C	-0.39161400	-1.07855100	-0.24666100

C	0.69873600	-1.83360500	0.18295400
C	1.94937500	-1.59335000	-0.35919200
H	1.32938500	0.76993900	-2.49491900
H	-0.95162400	0.47965400	-1.64565500
H	0.57283300	-2.58633800	0.95583300
H	2.80543500	-2.16937600	-0.01403500
N	2.18966200	-0.67723700	-1.29986000
C	-1.70123400	-1.27467000	0.37670800
H	-1.77772600	-2.14713700	1.02479100
C	-2.75245300	-0.47108100	0.23703400
H	-3.68710400	-0.68276700	0.74596900
H	-2.72474500	0.42590500	-0.37663600

Adenine

C	1.71722600	1.67844300	1.12474200
N	1.60877300	0.36639200	0.96085700
C	0.33317800	0.00465800	0.77331800
C	-0.76767300	0.85081300	0.75755400
C	-0.51055200	2.21774900	0.94175200
N	0.76065200	2.61106400	1.12611300
H	2.72669700	2.05495500	1.27933600
N	-1.93398500	0.14598400	0.53603000
N	-0.17660900	-1.24757600	0.57473400
H	0.34985300	-2.09663500	0.33223600
C	-1.52464400	-1.09058800	0.42085000
H	-2.16296700	-1.94182200	0.22641800
N	-1.48730700	3.13863700	0.95327800
H	-2.42162300	2.87933100	0.67445300
H	-1.23404700	4.11574800	0.94952600

Dicyclohexylamine

C	3.21411000	-1.70307800	1.31336500
C	2.85342200	-1.29572100	-0.12008600
C	1.59252200	-0.42420800	-0.18490000
C	0.61042400	-0.80542100	0.92153600
C	1.23982800	-0.49893200	2.28551100
C	2.76540300	-0.62904400	2.28906100
H	2.71953700	-2.64926700	1.57505300
H	3.69139100	-0.75050700	-0.57192100
H	1.84302600	0.64356800	-0.07274700
H	0.96441500	0.52660500	2.57027900
H	3.21927800	0.32724600	1.99227900
N	-0.71348900	-0.18717900	0.87019200
H	-0.58870500	0.81977400	0.99985600
C	-2.95494600	-0.57614200	-0.08048300

C	-3.83907200	-0.37399500	-1.31891200
C	-3.07499200	-0.70675000	-2.59100600
C	-1.88014600	0.24090000	-2.76325400
C	-1.31346600	0.70337300	-1.41648900
C	-1.45941900	-0.40073800	-0.37270800
H	-3.23428000	0.15174400	0.69279700
H	-4.74971100	-0.97786000	-1.24050200
H	-2.72527100	-1.74704000	-2.53317700
H	-1.09117800	-0.26398300	-3.33604900
H	-0.26975700	1.01450100	-1.52866400
H	-1.08101500	-1.33623800	-0.81449300
H	-1.85795400	1.58734600	-1.05120300
H	-2.17966800	1.11557400	-3.35386600
H	-3.73160000	-0.65529900	-3.46649200
H	-4.17203200	0.67190100	-1.37403500
H	-3.11828700	-1.57074400	0.35461000
H	0.45037800	-1.89387400	0.84232100
H	1.12850900	-0.53213200	-1.17139600
H	2.70757500	-2.19472500	-0.73189200
H	4.29078800	-1.88733600	1.39725700
H	3.12595200	-0.83663200	3.30241400
H	0.79544500	-1.16034400	3.04012900

VIM-ReO4− Complex:

Re	1.668000	0.831000	-2.028000
O	2.658000	1.161000	-3.444000
O	0.940000	2.328000	-1.428000
O	0.408000	-0.336000	-2.451000
O	2.686000	0.139000	-0.767000
C	-0.033000	-1.811000	0.505000
C	1.051000	-0.828000	2.049000
C	0.066000	0.037000	1.689000
N	-0.644000	-0.607000	0.695000
H	-0.400000	-2.512000	-0.234000
H	1.809000	-0.684000	2.806000
H	-0.203000	1.018000	2.048000
N	0.985000	-1.989000	1.306000
C	-1.731000	-0.138000	-0.062000
H	-2.136000	-0.899000	-0.723000

C	-2.228000	1.087000	0.002000
H	-3.078000	1.349000	-0.616000
H	-1.818000	1.854000	0.651000

AD-ReO4− Complex:

Re	0.952000	-2.744000	-2.332000
O	1.067000	-3.507000	-0.731000
O	-0.748000	-2.456000	-2.706000
O	1.785000	-1.187000	-2.308000
O	1.668000	-3.793000	-3.554000
C	1.717000	1.678000	1.125000
N	1.609000	0.366000	0.961000
C	0.333000	0.005000	0.773000
C	-0.768000	0.851000	0.758000
C	-0.511000	2.218000	0.942000
N	0.761000	2.611000	1.126000
H	2.727000	2.055000	1.279000
N	-1.934000	0.146000	0.536000
N	-0.177000	-1.248000	0.575000
H	0.350000	-2.097000	0.332000
C	-1.525000	-1.091000	0.421000
H	-2.163000	-1.942000	0.226000
N	-1.487000	3.139000	0.953000
H	-2.422000	2.879000	0.674000
H	-1.234000	4.116000	0.950000

CY-ReO4− Complex:

Re	0.634000	-1.875000	2.863000
O	1.432000	-2.431000	1.380000
O	-1.082000	-1.594000	2.554000
O	0.817000	-3.107000	4.108000
O	1.396000	-0.381000	3.411000
C	-0.269000	1.939000	-0.191000
C	0.135000	0.661000	-0.045000

C	-0.026000	-0.194000	-1.181000
C	-0.918000	1.514000	-2.475000
H	-0.208000	2.678000	0.599000
H	0.556000	0.296000	0.884000
N	-0.787000	2.356000	-1.370000
H	-1.090000	3.316000	-1.486000
N	-0.528000	0.228000	-2.339000
O	-1.385000	1.981000	-3.516000
N	0.339000	-1.474000	-1.082000
H	0.751000	-1.843000	-0.222000
H	0.233000	-2.069000	-1.891000

DCHA-ReO4− Complex:

Re	1.372000	3.647000	1.133000
O	2.394000	2.940000	2.386000
O	1.091000	5.360000	1.471000
O	2.161000	3.482000	-0.439000
O	-0.195000	2.825000	1.097000
C	3.214000	-1.703000	1.313000
C	2.853000	-1.296000	-0.120000
C	1.593000	-0.424000	-0.185000
C	0.610000	-0.805000	0.922000
C	1.240000	-0.499000	2.286000
C	2.765000	-0.629000	2.289000
H	2.720000	-2.649000	1.575000
H	3.691000	-0.751000	-0.572000
H	1.843000	0.644000	-0.073000
H	0.964000	0.527000	2.570000
H	3.219000	0.327000	1.992000
N	-0.713000	-0.187000	0.870000
H	-0.589000	0.820000	1.000000
C	-2.955000	-0.576000	-0.080000
C	-3.839000	-0.374000	-1.319000
C	-3.075000	-0.707000	-2.591000

C	-1.880000	0.241000	-2.763000
C	-1.313000	0.703000	-1.416000
C	-1.459000	-0.401000	-0.373000
H	-3.234000	0.152000	0.693000
H	-4.750000	-0.978000	-1.241000
H	-2.725000	-1.747000	-2.533000
H	-1.091000	-0.264000	-3.336000
H	-0.270000	1.015000	-1.529000
H	-1.081000	-1.336000	-0.814000
H	-1.858000	1.587000	-1.051000
H	-2.180000	1.116000	-3.354000
H	-3.732000	-0.655000	-3.466000
H	-4.172000	0.672000	-1.374000
H	-3.118000	-1.571000	0.355000
H	0.450000	-1.894000	0.842000
H	1.129000	-0.532000	-1.171000
H	2.708000	-2.195000	-0.732000
H	4.291000	-1.887000	1.397000
H	3.126000	-0.837000	3.302000
H	0.795000	-1.160000	3.040000

DIBA-ReO4− Complex:

Re	-0.722000	2.743000	1.574000
O	-1.323000	4.132000	2.494000
O	0.351000	1.799000	2.613000
O	-2.084000	1.742000	1.042000
O	0.202000	3.299000	0.166000
N	0.086000	-0.945000	-0.464000
H	-0.769000	-0.553000	-0.069000
C	1.072000	-0.983000	0.608000
H	2.018000	-1.350000	0.184000
H	1.285000	0.033000	0.995000
C	0.679000	-1.873000	1.775000
H	0.634000	-2.910000	1.406000

C	0.529000	-0.036000	-1.515000
H	1.485000	-0.413000	-1.905000
H	0.741000	0.975000	-1.110000
C	-0.451000	0.108000	-2.666000
H	-0.604000	-0.887000	-3.111000
C	-0.680000	-1.510000	2.356000
H	-1.498000	-1.665000	1.644000
H	-0.899000	-2.123000	3.237000
H	-0.697000	-0.458000	2.669000
C	1.750000	-1.778000	2.851000
H	1.549000	-2.463000	3.682000
H	2.745000	-2.010000	2.455000
H	1.784000	-0.760000	3.262000
C	-1.796000	0.651000	-2.206000
H	-2.466000	0.804000	-3.059000
H	-2.307000	-0.021000	-1.509000
H	-1.662000	1.620000	-1.705000
C	0.146000	1.025000	-3.721000
H	1.120000	0.662000	-4.070000
H	-0.512000	1.114000	-4.591000
H	0.292000	2.033000	-3.310000

DOA-ReO4− Complex:

Re	0.036000	-1.499000	2.352000
O	0.015000	-2.273000	3.942000
O	-0.166000	-2.742000	1.106000
O	-1.256000	-0.302000	2.225000
O	1.579000	-0.666000	2.104000
N	0.545000	0.677000	-0.686000
H	0.852000	0.294000	0.212000
C	0.135000	-0.459000	-1.502000
H	-0.221000	-0.086000	-2.474000
H	-0.729000	-0.985000	-1.047000
C	1.261000	-1.444000	-1.713000

H	2.080000	-0.953000	-2.258000
H	1.671000	-1.735000	-0.733000
C	0.824000	-2.688000	-2.462000
H	0.372000	-2.399000	-3.424000
H	0.031000	-3.196000	-1.892000
C	1.965000	-3.657000	-2.710000
H	2.765000	-3.142000	-3.266000
H	2.408000	-3.949000	-1.745000
C	1.549000	-4.902000	-3.471000
H	1.092000	-4.608000	-4.429000
H	0.762000	-5.428000	-2.909000
C	2.702000	-5.853000	-3.736000
H	3.488000	-5.326000	-4.299000
H	3.163000	-6.144000	-2.779000
C	2.294000	-7.102000	-4.496000
H	1.830000	-6.810000	-5.448000
H	1.514000	-7.631000	-3.929000
C	3.463000	-8.033000	-4.757000
H	3.157000	-8.935000	-5.297000
H	4.236000	-7.535000	-5.355000
H	3.931000	-8.351000	-3.818000
C	-0.585000	1.551000	-0.407000
H	-0.886000	2.054000	-1.338000
H	-1.466000	0.967000	-0.079000
C	-0.261000	2.575000	0.659000
H	0.254000	2.070000	1.490000
H	0.447000	3.318000	0.264000
C	-1.500000	3.265000	1.199000
H	-2.064000	3.723000	0.371000
H	-2.165000	2.504000	1.636000
C	-1.190000	4.316000	2.249000
H	-0.548000	3.870000	3.025000
H	-0.599000	5.127000	1.797000
C	-2.431000	4.893000	2.904000

H	-3.087000	5.326000	2.132000
H	-3.007000	4.076000	3.366000
C	-2.122000	5.945000	3.953000
H	-1.435000	5.520000	4.702000
H	-1.578000	6.781000	3.486000
C	-3.358000	6.480000	4.655000
H	-4.048000	6.895000	3.907000
H	-3.892000	5.644000	5.128000
C	-3.028000	7.534000	5.694000
H	-2.523000	8.394000	5.237000
H	-3.923000	7.907000	6.202000
H	-2.355000	7.132000	6.461000

DMAC-ReO4− Complex:

Re	-1.592000	-2.223000	-0.730000
O	-0.844000	-2.371000	0.864000
O	-2.349000	-0.628000	-0.850000
O	-0.343000	-2.398000	-1.970000
O	-2.828000	-3.473000	-0.931000
N	0.366000	1.172000	0.141000
C	1.216000	0.311000	-0.470000
O	1.407000	0.365000	-1.689000
C	-0.182000	0.964000	1.468000
H	-0.233000	1.917000	2.002000
H	-1.200000	0.557000	1.401000
H	0.429000	0.279000	2.053000
C	-0.387000	2.090000	-0.695000
H	-0.801000	2.879000	-0.063000
H	0.263000	2.545000	-1.443000
H	-1.212000	1.580000	-1.207000
C	1.938000	-0.695000	0.377000
H	1.243000	-1.473000	0.712000
H	2.717000	-1.156000	-0.232000

H	2.391000	-0.243000	1.263000

4-VP-ReO4− Complex:

Re	0.696000	2.594000	1.176000
O	0.859000	3.862000	2.394000
O	0.836000	1.018000	1.967000
O	-0.891000	2.703000	0.398000
O	1.957000	2.768000	-0.052000
C	1.135000	0.028000	-1.724000
C	-0.151000	-0.134000	-1.245000
C	-0.392000	-1.079000	-0.247000
C	0.699000	-1.834000	0.183000
C	1.949000	-1.593000	-0.359000
H	1.329000	0.770000	-2.495000
H	-0.952000	0.480000	-1.646000
H	0.573000	-2.586000	0.956000
H	2.805000	-2.169000	-0.014000
N	2.190000	-0.677000	-1.300000
C	-1.701000	-1.275000	0.377000
H	-1.778000	-2.147000	1.025000
C	-2.752000	-0.471000	0.237000
H	-3.687000	-0.683000	0.746000
H	-2.725000	0.426000	-0.377000
